# Supplementary material for: 7-Hydroxycoumarin Induces Vasorelaxation in Animals with Essential Hypertension: Focus on Potassium Channels and Intracellular Ca2+ Mobilization
Source: Molecules. 2022 Oct 28;27(21):7324. doi: 10.3390/molecules27217324 (PMC9655823; doi:10.3390/molecules27217324)
Supplement: Supplementary file 1 [file molecules-27-07324-s001.zip › molecules-1975535-supplementary.pdf]

Supplementary Material

7-hydroxycoumarin Induces Vasorelaxation in Animals with Essential Hypertension: Focus on Potassium Channels and Intracellular Ca^{2+} Mobilization

Rafael L. C. Jesus [1], Isnar L.P. Silva [1], Fênix A. Araújo [2], Raiana A. Moraes [2], Liliane B. Silva [1], Daniele S. Brito [1], Gabriela B.C. Lima [1], Quiara L. Alves [1] and Darizy F. Silva [1,2,*]

[1] Laboratory of Cardiovascular Physiology and Pharmacology, Federal University of Bahia, Salvador 40110-902, Brazil
[2] Gonçalo Moniz Institute, Oswaldo Cruz Foundation—FIOCRUZ, Salvador 40296-710, Brazil
* Correspondence: darizy@gmail.com (D.F.S); Tel.: +55-719-88-52-34-36

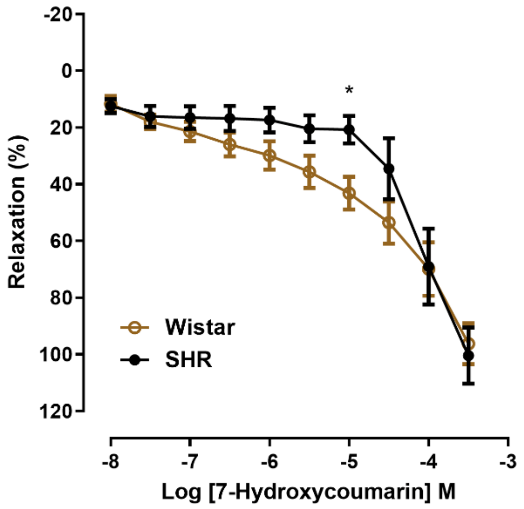

Figure S1. Vasorelaxation effects of 7-HC in the superior mesenteric artery from wistar and SHR rats**.** Results are expressed as mean ± S.E.M. Statistical analysis was performed using unpaired Student's t-tests. *$p < 0.05$ versus wistar.

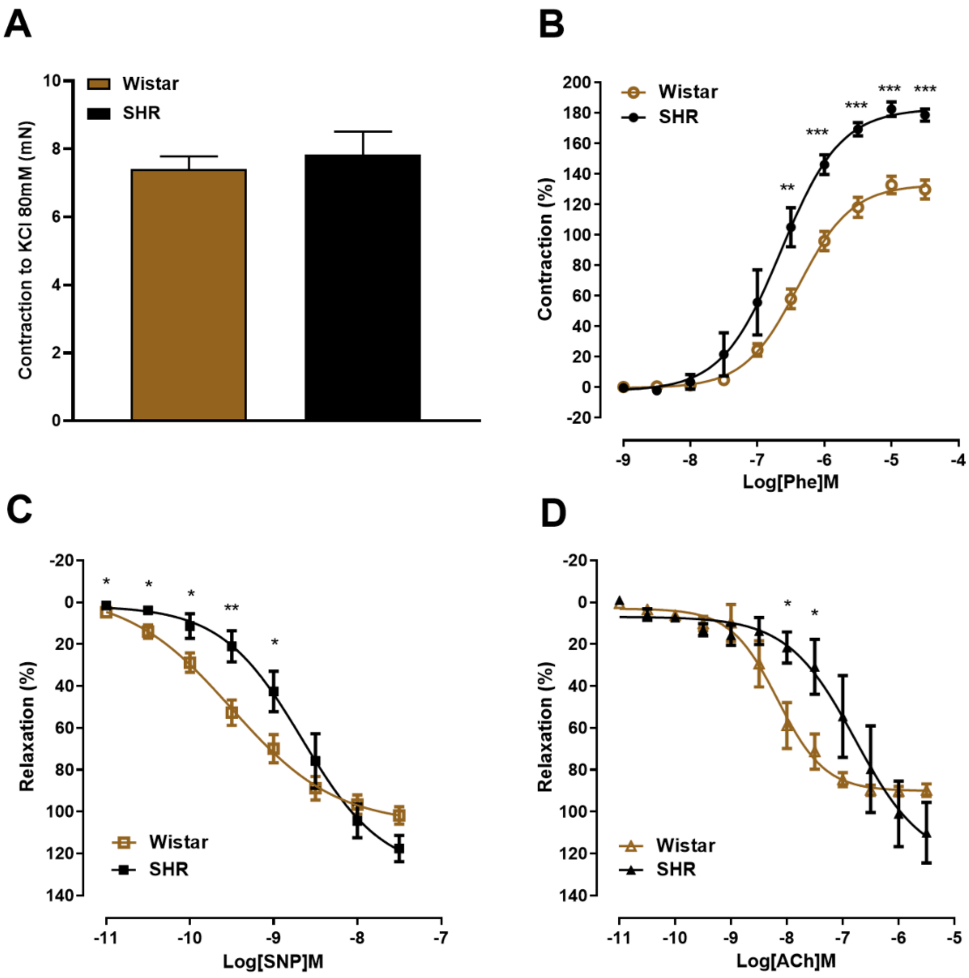

Figure S2. Vascular abnormalities in superior mesenteric artery from hypertensive compared to normotensive rats. **A**) Bar graphs showing contraction induced by Tyrode's solution containing KCl 80 mM. **B**) Contraction responses induced by Phe (10^{-9} to 3×10^{-5} M, cumulatively). **C**) Relaxation responses induced by SNP (10^{-11} to 3×10^{-8} M, cumulatively). **D**) Relaxation responses induced by Ach (10^{-11} to 3×10^{-6} M, cumulatively). Results are expressed as mean ± S.E.M. Statistical analysis was performed using unpaired Student's t-tests. *$p < 0.05$, **$p < 0.01$ or ***$p < 0.001$ versus wistar.
